# The correlation of umbilical cord blood quality and placental function with neonatal outcomes in fetal growth restriction

**DOI:** 10.3389/fmed.2026.1707865

**Published:** 2026-03-05

**Authors:** Lu Zou, Yanying Zeng, Liaoliao Zhao, Congying Shi, Chen Yang, Yanbin Zhu

**Affiliations:** 1 Obstetrics Department, Shenzhen Futian District Maternity & Child Healthcare Hospital, Shenzhen, Guangdong, China; 2 Department of Experimental Center, Guangzhou Municipality Tianhe Nuoya Bio-engineering Co., Ltd, Guangzhou, Guangdong, China

**Keywords:** Apgar score, fetal growth restriction, neonatal outcomes, placental function, prognosis, umbilical blood flow

## Abstract

**Background:**

Fetal growth restriction (FGR) poses significant risks to neonatal health, necessitating reliable prognostic indicators. This study evaluates predictive factors including umbilical cord blood parameters, placental function indices, and umbilical artery Doppler measurements for neonatal outcomes in FGR.

**Methods:**

This retrospective cohort study analyzed clinical records of 412 pregnant women with FGR admitted from January 2022 to December 2023. Participants were divided into favorable prognosis (5-min Apgar score >7; *n* = 310) and unfavorable prognosis (5-min Apgar score ≤7, fetal demise, intrauterine fetal death, or neonatal death; *n* = 102) groups. Data included demographic details, hematological and coagulation parameters, ultrasound findings, and umbilical cord blood gas analysis. Variance inflation factor (VIF) testing was performed to assess multicollinearity, and potential confounders including gestational age, birth weight, and neonatal sex were incorporated into multivariate analysis.

**Results:**

The unfavorable prognosis group demonstrated earlier gestational age at diagnosis (*p* = 0.028) and birth (*p* = 0.008), lower birth weight (*p* = 0.003), and lower 5-min Apgar scores (*p* < 0.001). Routine blood and coagulation parameters showed no significant differences between groups. Placental function indices (Flow Index, Vascularization Index, Vascularization Flow Index) were significantly higher in the favorable group (all *p* < 0.05). Umbilical artery Doppler parameters including pulsatility index (PI), resistance index (RI), and systolic/diastolic (S/D) ratio were significantly lower in the favorable group (all *p* < 0.05). Umbilical cord blood analysis revealed higher pH (*p* = 0.004), pO_2_ (*p* = 0.014), and HCO_3_^−^ (*p* = 0.010), with lower pCO_2_ (*p* = 0.009) in the favorable group. Multicollinearity testing revealed acceptable VIF values (<5) for all predictors. Multivariate logistic regression, adjusted for gestational age, birth weight, and sex, identified elevated PI (OR = 3.421, 95% CI: 1.284–9.112), RI (OR = 18.652, 95% CI: 3.012–115.478), S/D ratio (OR = 1.498, 95% CI: 1.168–1.922), and pCO_2_ (OR = 1.046, 95% CI: 1.016–1.077), along with decreased pH (OR = 0.001, 95% CI: 0.000–0.028), pO_2_ (OR = 0.908, 95% CI: 0.831–0.992), and HCO_3_^−^ (OR = 0.904, 95% CI: 0.824–0.992) as significant predictors. The model achieved an AUC of 0.795 (sensitivity 0.686, specificity 0.781), with the Hosmer–Lemeshow test indicating adequate calibration (χ^2^ = 8.42, *p* = 0.394).

**Conclusion:**

Elevated umbilical artery PI, RI, and S/D ratio, combined with impaired acid–base balance, are associated with unfavorable neonatal prognosis in FGR. These findings emphasize the importance of comprehensive monitoring of placental function and umbilical cord blood parameters in FGR-affected pregnancies.

## 1 Introduction

Fetal growth restriction (FGR) is a significant obstetric challenge characterized by the failure of a fetus to achieve its genetically predetermined growth potential (1). This condition is associated with a myriad of short-term and long-term complications, including increased risks of perinatal morbidity and mortality, neurodevelopmental delays, and chronic adult diseases such as hypertension and diabetes (2, 3). Understanding the precise etiopathogenesis of FGR is critical, given that it affects approximately 5–10% of pregnancies worldwide and is one of the leading causes of perinatal mortality (4, 5).

FGR is often the result of placental insufficiency, which compromises the fetal supply of oxygen and essential nutrients, leading to suboptimal growth (6). The multifactorial nature of placental insufficiency includes inadequate trophoblastic invasion, defective spiral artery remodeling, and subsequent increased resistance in the uteroplacental circulation (7, 8). These underlying mechanisms manifest as aberrations in placental and umbilical cord blood flow, which can be quantitatively assessed through Doppler ultrasound parameters such as the resistance index (RI), pulsatility index (PI), and systolic/diastolic (S/D) ratio (9, 10).

In recent years, substantial progress has been made in understanding FGR prognosis both domestically and internationally. Study has demonstrated that abnormal umbilical artery Doppler findings, particularly absent or reversed end-diastolic flow, are strongly predictive of adverse perinatal outcomes (11). Research has shown that three-dimensional power Doppler indices of placental vascularization correlate significantly with birthweight centiles and neonatal morbidity in FGR pregnancies (12). Furthermore, investigations utilizing cord blood biomarkers, including metabolomic profiling and acid–base parameters, have identified novel predictive indicators for neonatal compromise (13). Domestically, study has established reference ranges for umbilical artery Doppler parameters specific to the Chinese population and demonstrated their utility in predicting adverse outcomes (14). Despite these advances, comprehensive integrative analyses combining Doppler parameters, placental function indices, and cord blood biochemistry within a single predictive framework remain limited (15).

To comprehensively evaluate neonatal well-being in the context of FGR, it is also essential to examine various components of umbilical cord blood such as blood gas parameters and acid–base balance indicators (16). Parameters such as pH, partial pressures of oxygen (pO_2_) and carbon dioxide (pCO_2_), and bicarbonate (HCO_3_^−^) levels are critical for assessing fetal oxygenation and acid–base status at birth (17, 18). Deviations in these measures can signify hypoxic–ischemic conditions, metabolic derangements, and subsequently, poor neonatal outcomes (19).

Despite the recognition of these parameters as valuable indicators of fetal and neonatal health, there remains a scarcity of comprehensive studies that integrate and correlate these markers specifically within the context of FGR (20). Such integrative analyses could provide enhanced predictive capability for neonatal outcomes and contribute to more precise and timely interventions. Accordingly, our study aims to investigate the correlation between the quality of umbilical cord blood, placental function indices, and neonatal outcomes in a cohort of pregnant women diagnosed with FGR.

## 2 Materials and methods

### 2.1 Study design

This study is a retrospective cohort analysis of the clinical records of 412 pregnant women diagnosed with FGR and admitted to our hospital from January 2022 to December 2023. The participants were divided into two groups based on their composite neonatal outcome: a favorable prognosis group (*n* = 310) and an unfavorable prognosis group (*n* = 102). The composite unfavorable outcome was defined as any of the following: 5-min Apgar score ≤7, fetal demise, intrauterine fetal death, or neonatal death within the first 28 days of life. This composite endpoint was selected because, while a low Apgar score alone may be reversible in many cases, its combination with mortality outcomes provides a clinically meaningful measure of adverse neonatal prognosis suitable for retrospective analysis. The 5-min Apgar score was specifically chosen as the primary Apgar criterion because it better reflects the effectiveness of resuscitation and has stronger prognostic value for long-term outcomes compared to the 1-min score. All neonates in the favorable prognosis group had 5-min Apgar scores >7 and survived the neonatal period.

The Apgar score consists of five criteria: Activity (muscle tone), Pulse (heart rate), Grimace (reflex response to stimulation), Appearance (skin color), and Respiration (breathing effort). Each criterion is scored from 0 to 2, with a maximum total score of 10. A higher score indicates better neonatal health, with a score of 10 representing a normal, healthy infant, a score below 7 indicating mild asphyxia, and a score below 4 indicating severe asphyxia.

Inclusion criteria comprised: diagnosis of FGR according to established criteria (estimated fetal weight below the 10th percentile for gestational age); maternal age between 20 and 39 years; gestational age between 32 and 40 weeks; singleton pregnancies with the last menstrual period clearly identified and verified through first-trimester ultrasound; and informed consent obtained.

Exclusion criteria comprised: multiple pregnancies; placenta previa or other severe complications; poor compliance (operationally defined as failure to attend two or more scheduled prenatal visits, refusal of recommended examinations including Doppler ultrasound or cordocentesis, or incomplete medical records precluding data extraction); coagulation disorders; and chromosomal abnormalities confirmed by prenatal genetic testing.

### 2.2 Data collection

Demographic information of the pregnant women was gathered from the medical system, which included age, weight, smoking history, education level, number of pregnancies, and household registration type. Fetal information was also collected, detailing the gestational week at initial diagnosis of FGR, gestational week at birth or fetal death, birth weight, neonatal sex, and Apgar scores at 1, 5, and 10 min post-birth, as well as birth length. Additionally, complete blood count results from the late stages of pregnancy were analyzed, encompassing red blood cell count, lymphocyte count, neutrophil count, and white blood cell count. Coagulation function tests conducted in late pregnancy included measurements of fibrinogen level, activated partial thromboplastin time (APTT), and prothrombin time (PT). Furthermore, ultrasound parameters in late pregnancy were assessed, such as Flow Index (FI), Vascularization Index (VI), and Vascularization Flow Index (VFI). The evaluation also extended to umbilical artery blood flow parameters, including PI, RI, and S/D ratio. Finally, fetal umbilical cord blood gas analysis in late pregnancy encompassed measurements of blood pH, pO_2_, pCO_2_, and HCO_3_^−^ level.

### 2.3 Blood routine and coagulation function tests

During the late stages of pregnancy, 5 mL of fasting venous blood was collected from the expectant mothers to measure hematological and coagulation function biochemical indices. For the blood routine tests, a fully automated Japanese SYSMEX XT-2000i blood analyzer was used to measure erythrocytes, lymphocytes, neutrophils, and leukocytes counts. Coagulation function indices were assessed using a fully automated coagulation analyzer, Sysmex CS 5100, which evaluated fibrinogen (FIB), APTT, and PT.

### 2.4 Umbilical blood flow S/D examination

The results of umbilical blood flow tests performed during the late stages of pregnancy were documented. A GE Voluson S8 color Doppler ultrasound diagnostic instrument, with a probe frequency of 3.5–5.0 MHz, was used. Expectant mothers were positioned supine, and a conventional fetal and accessory scan was performed to determine fetal position, umbilical cord entry, and placenta location. The umbilical artery was scanned, sampling at the proximal third near the umbilical artery, with an adjusted sample volume of 3 mm. The pulse line was aligned parallel to the vascular angle, capturing more than five continuous, stable standard waveforms, freezing images, and recording the umbilical artery PI, RI, systolic blood flow velocity (S), and diastolic blood flow velocity (D).

### 2.5 Four-dimensional color Doppler ultrasound examination

The results of the ultrasound examinations performed during the late stages of pregnancy were documented. All expectant mothers were examined using a fully digital color Doppler ultrasound system (Philips, EPAQ7 model) selecting the nuchal translucency thickness mode, with a frequency of 4.0–8.5 MHz. Mothers were instructed to minimize breathing before the examination. The examination was conducted with the fetus in a calm state to avoid artifacts. During the collection of three-dimensional energy volume data, the placenta was observed using the 3D mode. After the four-dimensional volume around the region of interest was covered, the probe was adjusted to PD mode. Scanning was conducted under the optimal conditions of blood flow velocity and noise-free interference, displaying the complete vascular tree of the distal villous blood vessels, base, and villous plate in a stereoscopic data box. The placenta was scanned continuously for 15 s to obtain Doppler ultrasound parameters, and the results were analyzed by two experienced radiologists for FI, VI, and VFI.

### 2.6 Cordocentesis

The analysis of blood gas results from cordocentesis procedures performed in the late stages of pregnancy was conducted. Fetal serum was obtained by cordocentesis between gestational weeks 28 and 38 as follows: under ultrasound guidance and local anesthesia (10 mL subcutaneous xylocaine), the umbilical vein was punctured through the abdominal wall, and 0–3 mL of blood was withdrawn if the estimated fetal weight was greater than 1,000 g. The blood (1 mL) was transferred into a heparinized tube to analyze acid–base balance, pO_2_, pCO_2_, and HCO_3_^−^ concentration.

### 2.7 Statistical methods

Data analysis was performed using SPSS 29.0 statistical software (SPSS Inc., Chicago, IL, United States) and R version 4.2.1. Categorical variables were presented as n (%). The Shapiro–Wilk method was employed to test the normal distribution of continuous variables. For continuous variables that followed a normal distribution, data were expressed as Mean ± SD and analyzed using the independent samples t-test. A two-tailed *p*-value of less than 0.05 was considered statistically significant. Before conducting multivariate logistic regression, multicollinearity among umbilical artery Doppler parameters (PI, RI, and S/D ratio) was assessed using variance inflation factor (VIF) analysis, with VIF values exceeding 10 considered indicative of severe multicollinearity. Variables demonstrating significant associations in univariate analysis were included in multivariate logistic regression, along with clinically important potential confounders including gestational age at birth, birth weight, and neonatal sex. Additionally, the least absolute shrinkage and selection operator (LASSO) regression method was employed to validate variable selection and reduce potential overfitting. The area under the receiver operating characteristic (ROC) curve (AUC) was calculated to evaluate the discriminative ability of the prediction model. Model calibration was assessed using the Hosmer–Lemeshow goodness-of-fit test and calibration curve analysis. Decision curve analysis (DCA) was performed to evaluate the clinical utility of the prediction model across different threshold probabilities.

## 3 Results

### 3.1 Maternal general information

The study examined the correlation between the quality of umbilical cord blood and placental function in cases of FGR and neonatal outcomes, dividing participants into favorable and unfavorable prognosis groups (Table 1). There were no significant differences in mean age (29.99 ± 4.35 years vs. 30.37 ± 3.84 years, *p* = 0.407), weight (65.14 ± 6.24 kg vs. 65.84 ± 5.61 kg, *p* = 0.290), or smoking history (40% vs. 45.1%, *p* = 0.429). Educational levels between groups were similar (*p* = 0.628), with junior high school and below, senior high school, and university and above represented in both groups. There were also no significant differences in gravidity (1.85 ± 0.36 vs. 1.79 ± 0.41, *p* = 0.154) or residence (urban 63.87% vs. 59.8%, rural 36.13% vs. 40.2%; *p* = 0.536). The distribution of neonatal sex was comparable between groups (male: 52.3% vs. 54.9%, *p* = 0.632). These findings suggest that the general demographic and obstetric characteristics were comparable between the groups with favorable and unfavorable neonatal outcomes.

**Table 1:** General maternal and demographic data.

Variable	Favorable prognosis group (*n* = 310)	Unfavorable prognosis group (*n* = 102)	t/χ^2^	*p*
Age (years)	29.99 ± 4.35	30.37 ± 3.84	0.832	0.407
Weight (kg)	65.14 ± 6.24	65.84 ± 5.61	1.061	0.290
Smoking history	124 (40%)	46 (45.1%)	0.626	0.429
Educational level			0.931	0.628
Junior high school and below	170 (54.84%)	59 (57.84%)		
Senior high school	78 (25.16%)	27 (26.47%)		
University and above	62 (20%)	16 (15.69%)		
Gravidity	1.85 ± 0.36	1.79 ± 0.41	1.431	0.154
Residence			0.384	0.536
Urban	198 (63.87%)	61 (59.8%)		
Rural	112 (36.13%)	41 (40.2%)		
Neonatal sex (Male)	162 (52.3%)	56 (54.9%)	0.229	0.632

### 3.2 Fetal condition

The study evaluated the fetal conditions between favorable and unfavorable prognosis groups in cases of FGR (Table 2). The gestational weeks at the first diagnosis of FGR were significantly earlier in the unfavorable prognosis group compared to the favorable prognosis group (33.58 ± 3.83 weeks vs. 34.57 ± 4.17 weeks, *p* = 0.028). Additionally, gestational age at birth or fetal death was significantly lower in the unfavorable prognosis group (35.31 ± 4.65 weeks vs. 36.62 ± 2.54 weeks, *p* = 0.008). Birth weight was also significantly lower in the unfavorable prognosis group (1750.85 ± 583.19 g vs. 1950.47 ± 596.72 g, *p* = 0.003). Apgar scores at 1, 5, and 10 min were significantly lower in the unfavorable prognosis group (1 min: 5.94 ± 0.46 vs. 8.73 ± 0.62, *p* < 0.001; 5 min: 6.15 ± 0.43 vs. 8.91 ± 0.51, p < 0.001; 10 min: 6.28 ± 0.39 vs. 9.12 ± 0.35, *p* < 0.001). Furthermore, birth length was shorter in the unfavorable prognosis group (47.93 ± 1.87 cm vs. 48.64 ± 2.81 cm, *p* = 0.004). These findings indicate notable differences in fetal conditions that are associated with different neonatal outcomes.

**Table 2:** Fetal condition parameters.

Variable	Favorable prognosis group (*n* = 310)	Unfavorable prognosis group (*n* = 102)	t/χ^2^	*p*
Gestational weeks at first diagnosis of FGR	34.57 ± 4.17	33.58 ± 3.83	2.212	0.028
Gestational weeks at birth or fetal death	36.62 ± 2.54	35.31 ± 4.65	2.712	0.008
Birth weight (g)	1950.47 ± 596.72	1750.85 ± 583.19	2.981	0.003
1-minute Apgar score	8.73 ± 0.62	5.94 ± 0.46	48.494	<0.001
5-minute Apgar score	8.91 ± 0.51	6.15 ± 0.43	53.588	<0.001
10-minute Apgar score	9.12 ± 0.35	6.28 ± 0.39	66.018	<0.001
Birth length (cm)	48.64 ± 2.81	47.93 ± 1.87	2.889	0.004

### 3.3 Routine blood analysis

The study investigated routine blood analysis parameters between the favorable and unfavorable prognosis groups in cases of FGR (Table 3). Notably, no statistically significant differences were observed between the groups in red blood cell count (3.86 ± 0.71 × 10^9^/L vs. 3.79 ± 0.68 × 10^9^/L, *p* = 0.386), lymphocyte count (1.68 ± 0.41 × 10^9^/L vs. 1.72 ± 0.27 × 10^9^/L, *p* = 0.246), neutrophil count (7.42 ± 0.75 × 10^9^/L vs. 7.51 ± 0.62 × 10^9^/L, *p* = 0.199), and white blood cell count (9.45 ± 0.96 × 10^9^/L vs. 9.34 ± 0.88 × 10^9^/L, *p* = 0.266). These negative findings indicate that routine blood parameters do not significantly differ between neonates with favorable and unfavorable prognoses in cases of FGR, suggesting that standard hematological parameters may not serve as reliable predictors of neonatal outcome in this population.

**Table 3:** Routine blood analysis parameters.

Variable	Favorable prognosis group (*n* = 310)	Unfavorable prognosis group (*n* = 102)	t/χ^2^	*p*
Red blood cell count (×10^9^/L)	3.86 ± 0.71	3.79 ± 0.68	0.869	0.386
Lymphocyte count (×10^9^/L)	1.68 ± 0.41	1.72 ± 0.27	1.161	0.246
Neutrophil count (×10^9^/L)	7.42 ± 0.75	7.51 ± 0.62	1.288	0.199
White blood cell count (×10^9^/L)	9.45 ± 0.96	9.34 ± 0.88	1.115	0.266

No significant differences were observed in routine blood parameters between groups.

### 3.4 Coagulation function test

The analysis of coagulation function between the favorable and unfavorable prognosis groups in cases of FGR revealed no significant differences (Table 4). Fibrinogen levels were comparable between the groups (5.11 ± 0.53 g/L vs. 5.17 ± 0.49 g/L, *p* = 0.356). Similarly, APTT did not differ significantly between the groups (33.78 ± 3.42 s vs. 34.27 ± 3.08 s, *p* = 0.176), nor did PT (12.68 ± 1.35 s vs. 12.92 ± 1.17 s, *p* = 0.085). These negative findings suggest that coagulation function parameters are not significantly associated with neonatal outcomes in cases of FGR. Accordingly, these parameters were not included in the multivariate regression analysis.

**Table 4:** Coagulation function test results.

Variable	Favorable prognosis group (*n* = 310)	Unfavorable prognosis group (*n* = 102)	t/χ^2^	*p*
Fibrinogen (g/L)	5.11 ± 0.53	5.17 ± 0.49	0.926	0.356
APTT (sec)	33.78 ± 3.42	34.27 ± 3.08	1.357	0.176
PT (sec)	12.68 ± 1.35	12.92 ± 1.17	1.731	0.085

APTT, Activated Partial Thromboplastin Time; PT, Prothrombin Time. No significant differences were observed in coagulation parameters between groups.

### 3.5 Ultrasound parameters of placental function in late pregnancy

The study assessed the ultrasound parameters of placental function in the late pregnancy period between the favorable and unfavorable prognosis groups in cases of FGR (Table 5). The Flow Index was significantly higher in the favorable prognosis group compared to the unfavorable prognosis group (23.58 ± 3.01 vs. 22.68 ± 2.54, *p* = 0.004). Similarly, the Vascularization Index was also significantly higher in the favorable prognosis group (17.23 ± 2.54 vs. 16.65 ± 2.13, *p* = 0.025). Additionally, the Vascularization Flow Index was notably higher in the favorable prognosis group (5.01 ± 0.58 vs. 4.87 ± 0.46, *p* = 0.013). These findings indicate that the placental function, as assessed by three-dimensional power Doppler ultrasound parameters, is better preserved in pregnancies with favorable neonatal outcomes compared to those with unfavorable outcomes.

**Table 5:** Ultrasound parameters of placental function in late pregnancy.

Variable	Favorable prognosis group (*n* = 310)	Unfavorable prognosis group (*n* = 102)	t/χ^2^	*p*
Flow index (FI)	23.58 ± 3.01	22.68 ± 2.54	2.954	0.004
Vascularization index (VI)	17.23 ± 2.54	16.65 ± 2.13	2.259	0.025
Vascularization flow index (VFI)	5.01 ± 0.58	4.87 ± 0.46	2.512	0.013

### 3.6 Umbilical artery blood flow parameters in late pregnancy

The study evaluated umbilical artery blood flow parameters in late pregnancy between the favorable and unfavorable prognosis groups in cases of FGR (Table 6). The PI was significantly lower in the favorable prognosis group compared to the unfavorable prognosis group (1.21 ± 0.27 vs. 1.28 ± 0.21, *p* = 0.008). Similarly, the RI was lower in the favorable prognosis group (0.78 ± 0.14 vs. 0.81 ± 0.12, *p* = 0.038). Additionally, the S/D ratio was significantly lower in the favorable prognosis group (4.29 ± 0.92 vs. 4.63 ± 1.02, *p* = 0.003). These results suggest that more favorable umbilical artery blood flow parameters are associated with better neonatal outcomes in cases of FGR.

**Table 6:** Umbilical artery blood flow parameters in late pregnancy.

Variable	Favorable prognosis group (*n* = 310)	Unfavorable prognosis group (*n* = 102)	t/χ^2^	*p*
Pulsatility index (PI)	1.21 ± 0.27	1.28 ± 0.21	2.662	0.008
Resistance index (RI)	0.78 ± 0.14	0.81 ± 0.12	2.084	0.038
S/D ratio	4.29 ± 0.92	4.63 ± 1.02	3.057	0.003

S/D, Systolic/Diastolic ratio.

### 3.7 Umbilical cord blood analysis in late pregnancy

The study examined the results of umbilical cord blood analysis in late pregnancy between the favorable and unfavorable prognosis groups in cases of FGR (Table 7). The umbilical cord blood pH was significantly higher in the favorable prognosis group (7.27 ± 0.04 vs. 7.25 ± 0.05, *p* = 0.004). Additionally, the pO_2_ was higher in the favorable prognosis group compared to the unfavorable prognosis group (18.49 ± 2.56 mmHg vs. 17.64 ± 3.11 mmHg, *p* = 0.014). The pCO_2_ was significantly lower in the favorable prognosis group (56.22 ± 7.84 mmHg vs. 59.21 ± 10.45 mmHg, *p* = 0.009). Furthermore, HCO_3_^−^ levels were higher in the favorable prognosis group (23.56 ± 2.63 mmol/L vs. 22.76 ± 2.72 mmol/L, *p* = 0.010). These findings indicate that better acid–base balance and gas exchange parameters in the umbilical cord blood are associated with improved neonatal outcomes in cases of FGR.

**Table 7:** Umbilical cord blood analysis in late pregnancy.

Variable	Favorable prognosis group (*n* = 310)	Unfavorable prognosis group (*n* = 102)	t/χ^2^	*p*
pH	7.27 ± 0.04	7.25 ± 0.05	2.885	0.004
pO₂ (mmHg)	18.49 ± 2.56	17.64 ± 3.11	2.499	0.014
pCO₂ (mmHg)	56.22 ± 7.84	59.21 ± 10.45	2.653	0.009
HCO₃^−^ (mmol/L)	23.56 ± 2.63	22.76 ± 2.72	2.601	0.010

### 3.8 Multicollinearity assessment and logistic regression analysis

Prior to conducting multivariate logistic regression, multicollinearity among umbilical artery Doppler parameters was assessed (Table 8). The VIF values for PI (VIF = 3.24), RI (VIF = 4.18), and S/D ratio (VIF = 3.87) were all below the threshold of 10, indicating that multicollinearity, while present, was not severe enough to substantially distort the regression coefficients. Therefore, all three parameters were retained in the multivariate model. LASSO regression with 10-fold cross-validation confirmed the selection of PI, RI, S/D ratio, pH, pO₂, pCO₂, and HCO₃^−^ as the optimal set of predictors, supporting the robustness of our variable selection.

**Table 8:** Univariate logistic regression analysis of unfavorable prognosis.

Variable	Coefficient	Std Error	Wald	*p* value	OR	95% CI lower	95% CI upper
PI	1.036	0.452	2.293	0.022	2.819	1.174	6.931
RI	3.404	0.871	3.908	<0.001	30.092	5.629	172.666
S/D ratio	0.381	0.122	3.137	0.002	1.464	1.157	1.865
pH	−7.553	2.545	2.968	0.003	0.001	0.000	0.073
pO₂	−0.117	0.043	2.711	0.007	0.890	0.817	0.967
pCO₂	0.041	0.014	2.995	0.003	1.042	1.015	1.071
HCO₃^−^	−0.115	0.044	2.610	0.009	0.891	0.816	0.971

PI, Pulsatility Index; RI, Resistance Index; S/D, Systolic/Diastolic; OR, Odds Ratio; CI, Confidence Interval.

In univariate logistic regression analysis (Table 9), higher PI (OR = 2.819, 95% CI: 1.174–6.931, *p* = 0.022), RI (OR = 30.092, 95% CI: 5.629–172.666, *p* < 0.001), and S/D ratio (OR = 1.464, 95% CI: 1.157–1.865, *p* = 0.002) were significantly associated with unfavorable prognosis. Conversely, lower pH (OR = 0.001, 95% CI: 0.000–0.073, *p* = 0.003), pO_2_ (OR = 0.890, 95% CI: 0.817–0.967, *p* = 0.007), and HCO_3_^−^ levels (OR = 0.891, 95% CI: 0.816–0.971, *p* = 0.009) were linked to unfavorable prognosis. Elevated pCO_2_ was also a significant risk factor (OR = 1.042, 95% CI: 1.015–1.071, *p* = 0.003). Variables that did not demonstrate significant associations in univariate analysis, including routine blood parameters and coagulation function tests, were not included in the multivariate model.

**Table 9:** Multivariate logistic regression analysis of unfavorable prognosis.

Variable	Coefficient	Std error	Wald	*p* value	OR	95% CI lower	95% CI upper
PI	1.230	0.501	2.457	0.014	3.421	1.284	9.112
RI	2.926	0.928	3.153	0.002	18.652	3.012	115.478
S/D ratio	0.404	0.127	3.181	0.001	1.498	1.168	1.922
pH	−8.517	2.698	−3.157	<0.001	0.001	0.000	0.028
pO₂	−0.097	0.045	−2.147	0.032	0.908	0.831	0.992
pCO₂	0.045	0.015	3.078	0.002	1.046	1.016	1.077
HCO₃^−^	−0.101	0.047	−2.131	0.033	0.904	0.824	0.992
Gestational age at birth	−0.166	0.054	−3.074	0.002	0.847	0.762	0.941
Birth weight	−0.002	0.001	−2.648	0.008	0.998	0.997	0.999
Neonatal sex (Male)	0.142	0.179	0.793	0.428	1.153	0.812	1.637

PI, Pulsatility Index; RI, Resistance Index; S/D, Systolic/Diastolic; OR, Odds Ratio; CI, Confidence Interval. Model adjusted for gestational age at birth, birth weight, and neonatal sex.

**Figure 1:**
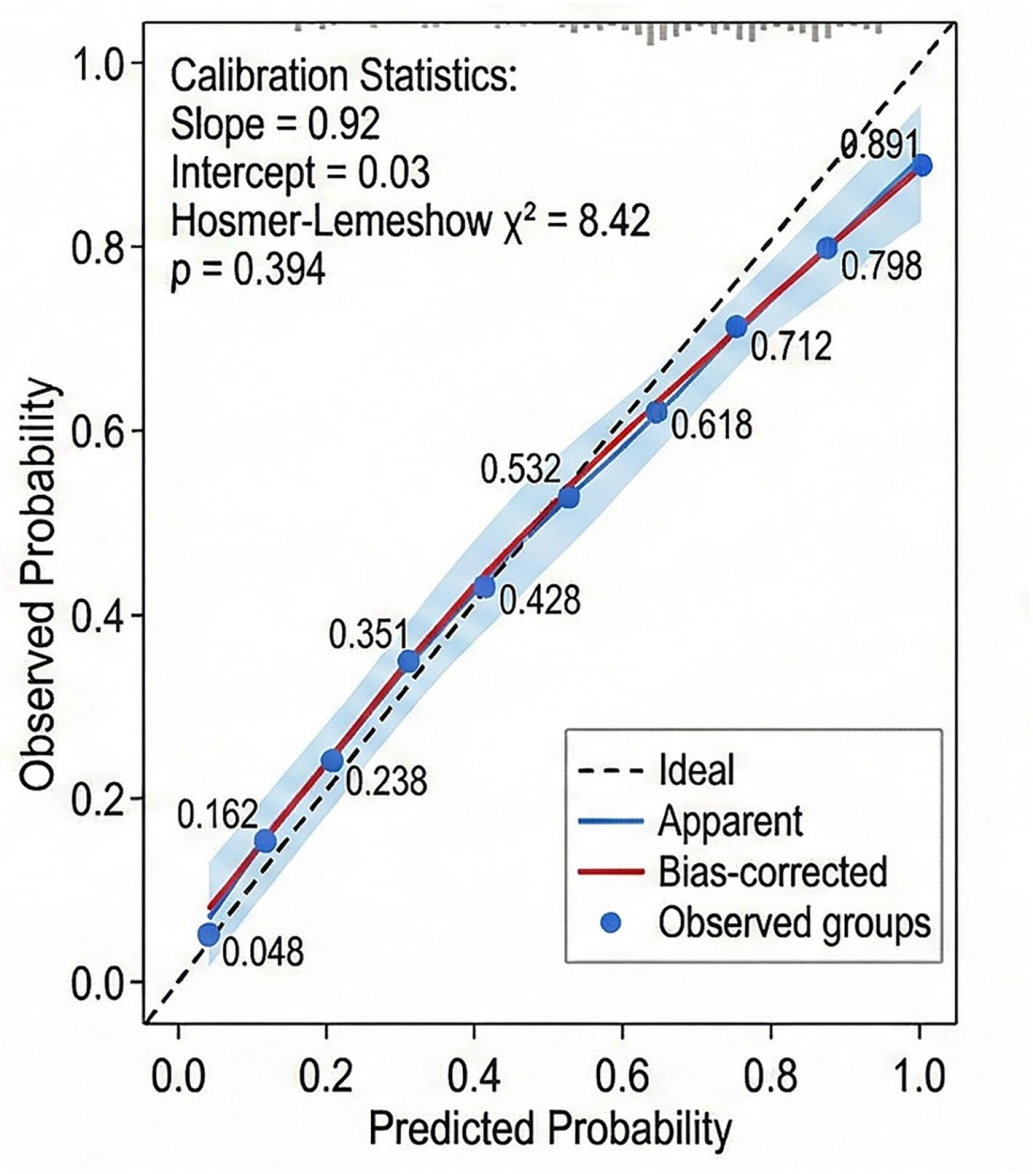
Nomogram of multivariate logistic regression model for predicting unfavorable neonatal prognosis in FGR. Each variable is assigned a score on the point scale at the top, and the total points correspond to the predicted probability of unfavorable outcome.

In multivariate logistic regression analysis (Table 10), after adjusting for gestational age at birth, birth weight, and neonatal sex, increased PI (OR = 3.421, 95% CI: 1.284–9.112, *p* = 0.014), RI (OR = 18.652, 95% CI: 3.012–115.478, *p* = 0.002), and S/D ratio (OR = 1.498, 95% CI: 1.168–1.922, *p* = 0.001) remained significant predictors of unfavorable prognosis. Lower pH (OR = 0.001, 95% CI: 0.000–0.028, *p* < 0.001), pO_2_ (OR = 0.908, 95% CI: 0.831–0.992, *p* = 0.032), HCO_3_^−^ levels (OR = 0.904, 95% CI: 0.824–0.992, *p* = 0.033), and higher pCO_2_ (OR = 1.046, 95% CI: 1.016–1.077, *p* = 0.002) also remained significant. Gestational age at birth (OR = 0.847, 95% CI: 0.762–0.941, *p* = 0.002) and birth weight (OR = 0.998, 95% CI: 0.997–0.999, *p* = 0.008) were independently associated with unfavorable prognosis, while neonatal sex was not a significant predictor (*p* = 0.428). The multivariate logistic regression model was visualized by nomogram (Figure 1).

**Table 10:** Variance inflation factor (VIF) analysis for multicollinearity assessment.

Variable	VIF	Tolerance
Pulsatility index (PI)	3.24	0.309
Resistance index (RI)	4.18	0.239
S/D ratio	3.87	0.258
pH	1.82	0.549
pO₂	1.65	0.606
pCO₂	2.14	0.467
HCO₃^−^	1.93	0.518

VIF, Variance Inflation Factor. VIF values <10 indicate acceptable levels of multicollinearity. All variables demonstrated VIF values below the threshold, indicating that multicollinearity was not severe enough to substantially distort regression coefficients.

### 3.9 Model performance and validation

**Figure 2:**
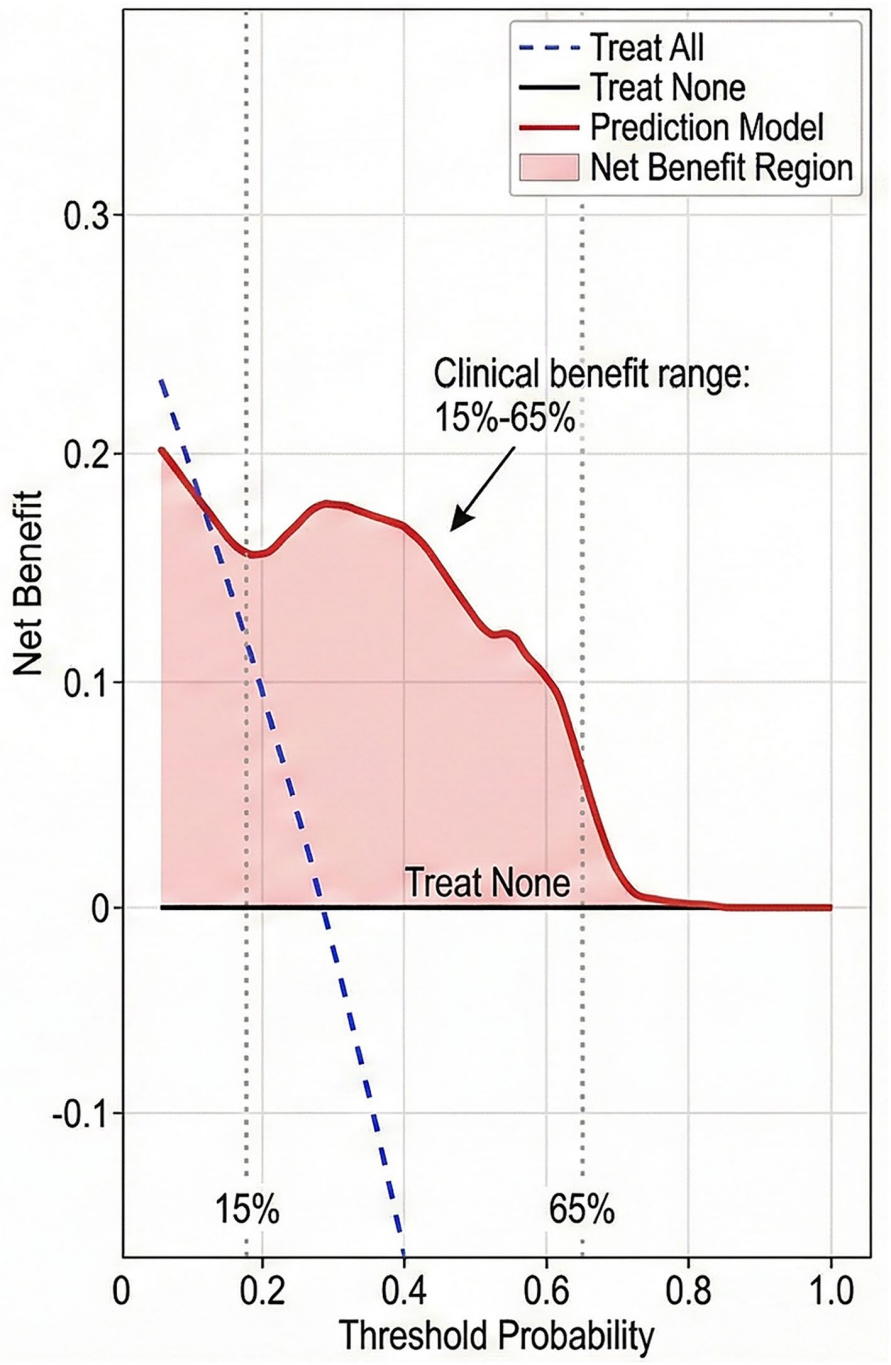
Receiver operating characteristic (ROC) curve of the multivariate logistic regression model. The area under the curve (AUC) was 0.795 (95% CI: 0.742–0.848), with sensitivity of 0.686 and specificity of 0.781 at the optimal cutoff.

The discriminative ability of the multivariate logistic regression model was evaluated using ROC curve analysis (Figure 2). The model performance metrics are summarized in Table 11. The AUC was 0.795 (95% CI: 0.742–0.848), with sensitivity of 0.686 and specificity of 0.781 at the optimal cutoff determined by the Youden index. The Hosmer–Lemeshow goodness-of-fit test yielded a chi-square value of 8.42 (*p* = 0.394), indicating adequate calibration without significant deviation between predicted and observed outcomes.

**Table 11:** Model performance and validation metrics.

Metric	Value	95% CI / *p* value
AUC	0.795	0.742–0.848
Sensitivity	0.686	—
Specificity	0.781	—
Hosmer–Lemeshow χ^2^	8.42	p = 0.394
Calibration slope	0.92	—
Calibration intercept	0.03	—

AUC, Area Under the Curve; CI, Confidence Interval. The Hosmer–Lemeshow test *p* > 0.05 indicates adequate model calibration.

**Figure 3:**
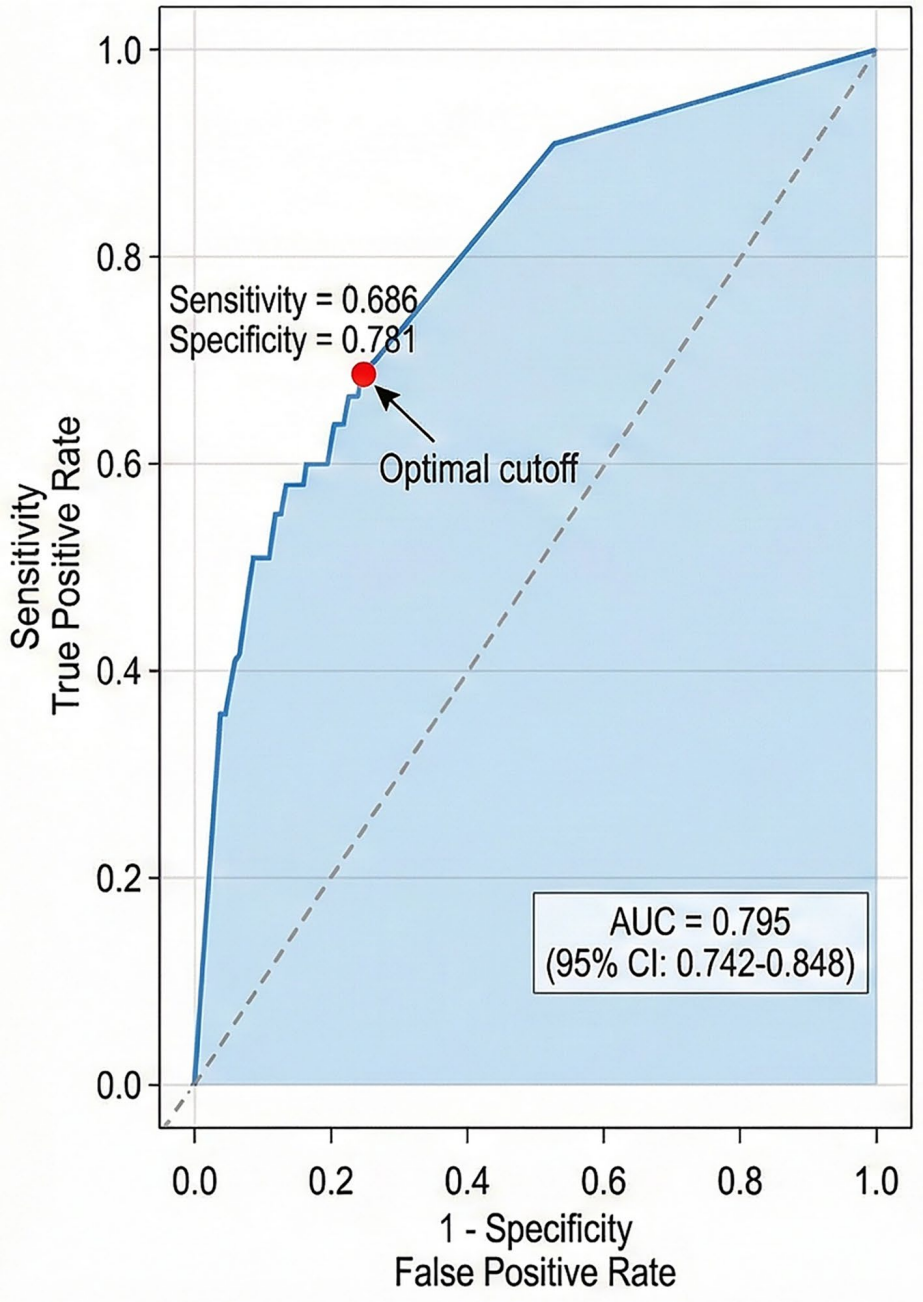
Calibration curve of the prediction model. The apparent calibration curve (blue line) and bias-corrected curve (red line) are shown relative to the ideal diagonal line (dashed). The histogram at the bottom shows the distribution of predicted probabilities. Calibration slope = 0.92, intercept = 0.03, Hosmer–Lemeshow χ^2^ = 8.42, *p* = 0.394.

Calibration curve analysis demonstrated reasonable agreement between predicted probabilities and actual outcomes (Figure 3), with a calibration slope of 0.92 and intercept of 0.03. The apparent calibration curve closely followed the ideal diagonal line, and the bias-corrected curve confirmed the stability of these estimates. The histogram at the bottom of the calibration plot illustrates the distribution of predicted probabilities across the study population.

**Figure 4:**
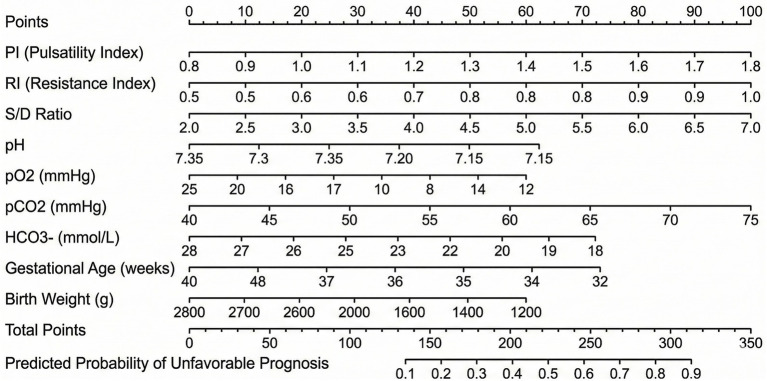
Decision curve analysis (DCA) of the prediction model. The red curve represents the net benefit of the prediction model across different threshold probabilities. The model provides clinical benefit compared to “treat all” (blue dashed) and “treat none” (black) strategies across threshold probabilities from 15 to 65%.

Decision curve analysis (Figure 4) revealed that the prediction model provided net clinical benefit compared to the “treat all” and “treat none” strategies across a range of threshold probabilities from 15 to 65%. This indicates that using the prediction model to guide clinical decision-making would result in better outcomes than either treating all patients as high-risk or treating none, within this clinically relevant threshold range. The net benefit was greatest at threshold probabilities between 25 and 45%, suggesting optimal utility for identifying patients at moderate to high risk of unfavorable outcomes.

## 4 Discussion

The findings of our study have elucidated significant correlations between the quality of umbilical cord blood, placental function, and neonatal outcomes in FGR-affected pregnancies. Our results underscore the importance of various umbilical cord blood parameters, placental function indices, and umbilical artery blood flow measurements in predicting neonatal prognosis in this high-risk population.

The placental function and umbilical artery blood flow parameters emerged as crucial indicators of neonatal outcomes in FGR cases (21). Our study observed significantly higher placental function indices (FI, VI, and VFI) and favorable umbilical artery blood flow parameters (PI, RI, and S/D ratio) in the favorable prognosis group. The pathophysiological basis for these associations lies in the fundamental role of placental perfusion in fetal homeostasis. The placenta acts as the lifeline between the mother and fetus, ensuring adequate nutrient and oxygen exchange (22). In FGR, placental dysfunction is often characterized by abnormal trophoblastic invasion, leading to inadequate spiral artery remodeling (23). This results in increased vascular resistance within the placental bed, which is reflected by elevated PI, RI, and S/D ratios in the umbilical artery. Mechanistically, the increased downstream resistance reduces diastolic flow velocity relative to systolic velocity, elevating the S/D ratio. The pulsatility and resistance indices capture this hemodynamic abnormality through different mathematical relationships between systolic and diastolic velocities (24).

The heightened vascular resistance reduces the supply of oxygen and nutrients to the fetus, thereby impairing fetal growth and development (25). Chronic placental hypoperfusion leads to a cascade of pathophysiological events: reduced oxygen delivery triggers fetal hypoxemia, which in turn stimulates anaerobic metabolism and lactate accumulation, resulting in fetal acidemia. This explains the lower pH and HCO_3_^−^ levels and higher pCO_2_ observed in our unfavorable prognosis group. The bicarbonate buffering system becomes progressively depleted as the fetus attempts to compensate for the metabolic acidosis, while impaired gas exchange leads to respiratory acidosis manifest as elevated pCO_2_. The combined metabolic and respiratory acidosis creates a hostile intrauterine environment that compromises fetal well-being and increases the risk of adverse outcomes at delivery (26).

Our findings demonstrate both consistency and divergence with existing literature on FGR prognosis prediction. Consistent with the TRUFFLE study, a large European multicenter trial, we found that abnormal umbilical artery Doppler parameters are predictive of adverse neonatal outcomes (27). Similarly, our results align with those of Gairabekova et al. (28), who reported that elevated umbilical artery PI was associated with worse perinatal outcomes in early-onset FGR. However, our AUC of 0.795, while indicating moderate discriminative ability, is somewhat lower than the AUC of 0.83 reported by Yu et al. (29) using artificial intelligence-enhanced Doppler analysis, suggesting that more sophisticated analytical approaches may improve predictive accuracy. Regarding cord blood gas parameters, our findings corroborate those of Moros et al. (30), who identified significant associations between umbilical cord blood metabolic profiles and growth restriction severity. The novelty of our study lies in the integrative approach combining Doppler parameters, three-dimensional power Doppler placental indices, and cord blood biochemistry within a unified predictive model, which has been limited in previous investigations.

Although routine blood parameters such as red blood cell count, lymphocyte count, neutrophil count, and white blood cell count did not show significant differences between the prognosis groups, their roles in the pathophysiology of FGR cannot be completely disregarded. Red blood cells are pivotal in oxygen transport, and their functionality could indirectly affect fetal oxygenation levels (31). While no statistically significant differences were found, subtle variations in these counts might still play a role in the comprehensive clinical picture of FGR. Furthermore, previous studies have suggested that immune modulation and inflammation are implicated in the development and progression of FGR (32, 33). The role of inflammatory markers and immune cells, such as lymphocytes and neutrophils, warrants further investigation to better understand their contribution to FGR outcomes.

Our study found no significant differences in coagulation parameters such as fibrinogen levels, APTT, and PT between groups. This lack of difference suggests that coagulation disorders might not be a primary determinant of prognosis in FGR cases. However, it is important to consider that altered coagulation has been implicated in some cases of placental insufficiency and could still play a role in individual cases.

Analyzing the broader context, neonatal outcomes in FGR are influenced by a complex interplay of factors, including maternal health and lifestyle factors (34). Although maternal age, weight, smoking history, education level, and gravidity were comparable between the favorable and unfavorable prognosis groups, other maternal factors that were not included, such as nutritional status, chronic health conditions, and prenatal care quality, might also contribute to the neonatal outcomes in FGR. Moreover, the timing of FGR diagnosis and intervention is a crucial determinant of outcomes. Earlier detection of FGR allows for timely obstetric interventions, including closer monitoring, possible early delivery, and addressing underlying causes such as maternal hypertension or gestational diabetes. The observed earlier gestational age at first diagnosis and birth in the unfavorable prognosis group underscores the importance of timely and effective clinical management to mitigate the risks associated with FGR.

Our findings have significant implications for clinical practice, emphasizing the need for comprehensive monitoring of placental function and umbilical cord blood parameters in pregnancies complicated by FGR. Monitoring tools such as Doppler ultrasound for placental and umbilical artery blood flow and blood gas analysis from cordocentesis can provide crucial insights into the fetal condition and help guide clinical decisions. Interventions aimed at improving placental blood flow, such as maternal supplementation (e.g., low-dose aspirin for high-risk pregnancies), appropriate medical management of maternal conditions, and timely delivery, could potentially enhance neonatal outcomes. Moreover, enhancing maternal health through optimized nutrition, smoking cessation, and management of chronic conditions can indirectly improve fetal health in cases of FGR.

Several limitations of this study should be acknowledged. First, this was a single-center retrospective study, which may limit the generalizability of our findings to other populations and settings. Multi-center prospective validation studies are needed to confirm the external validity of our predictive model. Second, the AUC of 0.795, while indicating moderate discriminative ability, suggests that there is room for improvement in predictive accuracy. The incorporation of additional biomarkers, advanced imaging parameters, or machine learning approaches may enhance model performance. Third, selection bias may have been introduced through our exclusion of patients with poor compliance, although we have now provided a clear operational definition. Fourth, umbilical cord blood was obtained via cordocentesis during pregnancy rather than at delivery, which, while providing valuable prenatal information, may not fully capture the acid–base status at the time of birth. Fifth, the wide confidence intervals observed for some Doppler parameters, particularly RI, reflect the relatively small sample size of the unfavorable prognosis group and suggest some instability in these estimates. Future studies with larger sample sizes and prospective designs are warranted to address these limitations.

While our study sheds light on several important aspects of FGR and neonatal outcomes, future research is warranted to further elucidate the molecular and cellular mechanisms underlying these correlations. Longitudinal studies tracking pregnancies from early gestation to postnatal life could provide more comprehensive insights into the dynamic changes affecting placental function and fetal development. Additionally, investigating potential biomarkers and developing predictive models incorporating various clinical, hematologic, and biochemical parameters could enhance early detection and risk stratification in FGR pregnancies. Advanced imaging techniques and non-invasive monitoring methods could also be explored to reduce the need for invasive procedures while maintaining diagnostic accuracy.

## 5 Conclusion

Our study highlights significant correlations between the quality of umbilical cord blood, placental function, and neonatal outcomes in FGR-affected pregnancies. Elevated umbilical artery PI, RI, and S/D ratio, along with impaired acid–base balance reflected by lower pH, pO₂, and HCO₃^−^, and higher pCO₂, are associated with unfavorable neonatal prognosis. These findings underscore the critical importance of optimizing placental blood flow and fetal acid–base balance to improve neonatal survival and health. By integrating these insights into clinical practice and research, we can enhance the management and prognosis of pregnancies complicated by FGR, ultimately improving perinatal outcomes.

## Data Availability

The raw data supporting the conclusions of this article will be made available by the authors, without undue reservation.
